# *In Vitro* Methods to Model Cardiac Mechanobiology in Health and Disease

**DOI:** 10.1089/ten.tec.2020.0342

**Published:** 2021-03-15

**Authors:** Ignasi Jorba, Dylan Mostert, Leon H.L. Hermans, Atze van der Pol, Nicholas A. Kurniawan, Carlijn V.C. Bouten

**Affiliations:** ^1^ Department of Biomedical Engineering, Eindhoven University of Technology, Eindhoven, The Netherlands.; ^2^ Institute for Complex Molecular Systems (ICMS), Eindhoven, The Netherlands.

**Keywords:** *cardiac *in vitro* models*, mechanobiology, cardiac (patho)physiology, multiscale cardiac mechanical properties, engineered heart tissue

## Abstract

*In vitro* cardiac modeling has taken great strides in the past decade. While most cell and engineered tissue models have focused on cell and tissue contractile function as readouts, mechanobiological cues from the cell environment that affect this function, such as matrix stiffness or organization, are less well explored. In this study, we review two-dimensional (2D) and three-dimensional (3D) models of cardiac function that allow for systematic manipulation or precise control of mechanobiological cues under simulated (patho)physiological conditions while acquiring multiple readouts of cell and tissue function. We summarize the cell types used in these models and highlight the importance of linking 2D and 3D models to address the multiscale organization and mechanical behavior. Finally, we provide directions on how to advance *in vitro* modeling for cardiac mechanobiology using next generation hydrogels that mimic mechanical and structural environmental features at different length scales and diseased cell types, along with the development of new tissue fabrication and readout techniques.

**Impact statement:**

Understanding the impact of mechanobiology in cardiac (patho)physiology is essential for developing effective tissue regeneration and drug discovery strategies and requires detailed cause–effect studies. The development of three-dimensional *in vitro* models allows for such studies with high experimental control, while integrating knowledge from complementary cell culture models and *in vivo* studies for this purpose. Complemented by the use of human-induced pluripotent stem cells, with or without predisposed genetic diseases, these *in vitro* models will offer promising outlooks to delineate the impact of mechanobiological cues on human cardiac (patho)physiology in a dish.

## Introduction

The human heart is of particular interest from a mechanobiological perspective since its tissues are under continuous mechanical stress while it cyclically contracts to pump blood throughout the body. At the cellular level, this results in an ongoing and dynamic mechanical perturbation of the cardiac cells, either by cellular contraction or triggered by the physical properties of the surrounding extracellular matrix (ECM). In particular, the physical forces in—and mechanical properties of—the cellular niche regulate a myriad of cellular responses, including migration, differentiation, division, and contractility.^1–5^

The fiber organization of the cardiac ECM, on the other hand, is essential for coordinated contraction.^6^ Breakdown of these mechanical and organizational features is a hallmark of cardiac pathophysiology. Following myocardial infarction (MI), for example, a large number of cardiomyocytes perish and are replaced by fibroblasts that transform this region into scar tissue. This process leads to an ongoing adverse ECM remodeling resulting in changes in the mechanical properties and a loss of the characteristic anisotropic tissue organization.^7^ To compensate for the loss of contractile force in the tissue, the remaining cardiomyocytes undergo hypertrophy.^8^

For over a century, *in vivo* animal models have been used as the primary model to study the biomechanical aspects of cardiac disease development and ECM remodeling. Although *in vivo* models are essential to monitor disease development and ECM changes over time and under complex physiological conditions—relevant for translation to the clinical setting—they have two important limitations.

First, the nature of the cardiovascular system differs among species. For instance, commonly-used small animals for cardiovascular research, like rodents, have smaller hearts, higher metabolism, and higher heart rates than humans. This has implications for comparing cellular electrophysiology, since repolarization times are shorter for rodent than for human cardiac tissue.^9^ In addition, rodents have thinner (i.e., single layer) cardiac walls with different mechanical strain patterns and ECM organization,^10^ impeding the translation of (adverse) ECM comparison to humans.

These differences have motivated the use of larger animals, like pigs, in cardiac pathophysiology. Still, these large animal models cannot avoid the second limitation of *in vivo* studies, namely that they do not allow precise control and systematic manipulation of mechanical and structural cues from the cardiac environment to understand the role of mechanobiology in cardiac (patho)physiology. The development of cardiac *in vitro* models with human-like tissue composition, organization, and mechanical behavior will allow such mechanistic understanding at the cellular and tissue scale.

The goal of any model is to have distinct experimental or predictive advantages while remaining as close as possible to the physiological reality. Isolated cardiac cells or cell monolayers seeded in standard culture dishes are not representative of the complex mechanical environment present in the heart. Cardiac *in vitro* tissue models try to close the gap between standard cell culture approaches and the complex heart physiology. Using a bottom-up approach, scientists engineered two-dimensional (2D) models to mimic ECM stiffness, ECM organization, and the mechanical stresses present in the (patho)physiologic tissue.^11^ These models have been fundamental in gaining knowledge of cardiac cell mechanobiology, interactions between neighboring cells, and electrophysiology. However, they do not mimic the more complex three-dimensional (3D) *in vivo* environment that integrates all mechanical stimuli present at a cellular level. Novel 3D *in vitro* models have been developed where cardiac cells show more physiological behavior such as self-assembly, ECM remodeling, and synchronized contraction.^12–14^

Based on these developments, substantial progress has been made to understand the effect of physical stimuli in cardiac (patho)physiology. However, cardiac *in vitro* models are still in their infancy regarding tissue mechanical function and integration of mechanobiological stimuli present in cardiac tissue, necessitating the development of more advanced *in vitro* models to mimic cardiac mechanobiology. The challenges for these next generation cardiac *in vitro* models hinge on three key issues: (1) characterizing the mechanical properties, ECM organization, and 3D stresses present in healthy and diseased cardiac tissue, (2) developing novel strategies to mimic and integrate these mechanical and organizational cues *in vitro*, and (3) integrating techniques to analyze cardiac function *in vitro*.

In this review, we first describe the complex cardiac mechanical environment across length scales from tissue to cell. Next, we review the state-of-the-art in cardiac cell models and their application in 2D and 3D *in vitro* models of cardiac mechanobiology. Finally, we provide directions to advance the modeling of cardiac mechanobiology *in vitro* taking into account outstanding challenges and limitations of current models.

## Multiscale Myocardial Mechanics and Organization: From Tissue to Cellular Microenvironment

**FIG. 1.**
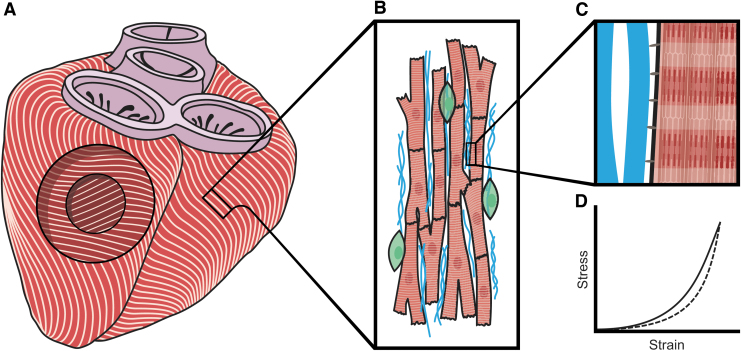
The mechanical and organizational environment of cardiac tissue at multiple length scales. **(A)** Differential layer organization of muscle fibers in the myocardium: subendocardial region (*inner layer*), middle myocardium region (*middle layer*), and subepicardial region (*outer layer*). **(B)** At the tissue level, cardiac cells (cardiomyocytes, *red*; fibroblasts, *green*) are anisotropically oriented along ECM fibers (*blue*). **(C)** Cardiomyocytes transmit the contraction force to the ECM by cell-ECM attachments (*gray*). **(D)** Nonlinear viscoelastic behavior is a characteristic of the passive mechanical properties of cardiac tissue, indicated by an increase in the tissue stiffness (i.e., the slope of stress–strain curve) with increasing tissue deformation and a loss of energy between mechanical loading (*solid line*) and unloading (*dashed line*) of the tissue. ECM, extracellular matrix.

Cardiac function is highly coupled to tissue organization and mechanics. In the left ventricle, muscle fibers are oriented helically throughout the myocardial wall layers with a gradual change in angle, layer to layer.^15,16^ In the innermost layer of the cardiac wall (subendocardial layer), fibers show a right-handed helical structure. Fibers in the middle layer are oriented circumferentially, while fibers in the outermost layer (subepicardial layer) are oriented in a left-handed helical arrangement (Fig. 1A). During systole, due to this helical organization, the myocardium rotates relative to the base to ensure complete blood ejection from the left ventricle.^17^

Due to dynamic ventricle contraction throughout the cardiac cycle, changes in tissue strain occur. During diastole, when the ventricle fills with blood, tension in the myocardium builds up until the end-diastolic pressure, referred as preload. During systole, tissue contraction overcomes the arterial systemic pressure or afterload, resulting in the blood's ejection from the ventricle. Overall, the organizational and mechanical environment of the myocardium consists of a complex 3D fiber organization under cyclic strain.

At the tissue level, each of the ventricular layers consists of an anisotropic array of cardiac cells in parallel alignment with the ECM fibers to ensure the coordinated contraction of the whole ventricle (Fig. 1B). The mechanical properties of the fibers are essential to ensure the proper force transmission through the tissue. In this sense, the ECM fibers are the primary determinants of the passive mechanical properties of the myocardium.^18^ Standard techniques to characterize passive material properties of the myocardium include compression and tension tests on freshly excised centimeter- and millimeter-scale tissue samples. Using such techniques it was demonstrated that myocardium displays nonlinear stress–strain behavior and loss/dissipation (viscoelasticity), a characteristic shared by almost all biological soft tissues.^19^

Myocardial viscoelasticity is higher along the mean-fiber direction than in the cross-fiber direction to withstand myocardial contraction and allow for a large preload.^20,21^ The nonlinear behavior by strain stiffening can be largely explained by the sequential alignment and strain of the protein fibers forming the ECM.^22^ Physiologically, it is advantageous for a tissue to become increasingly resistant to extension as it is deformed to prevent excessive deformations and tissue damage.^23^ In the myocardium, distensibility at low strains enables the correct blood filling of the relaxed ventricle until the end-diastolic volume is achieved at higher strains.

Cells attach to ECM by focal adhesions (Fig. 1C), which sense ECM fiber viscoelasticity. Studies measuring ECM mechanical properties using highly precise nanoscale techniques, including atomic force microscopy (AFM), capture the same nonlinear viscoelastic behavior showed at the tissue level but at the micrometer scale (Fig. 1D).^24–27^ Interestingly, myocardial ECM mechanical properties at the cell scale are around one order of magnitude higher than those at the tissue scale. This difference can be explained by the complex 3D structure forming the myocardium, highlighting the importance of understanding such a multiscale phenomenon.^28^

Under pathological conditions, for example after MI, the characteristic anisotropic 3D ECM structure and viscoelasticity are altered. The fibrotic scar produced to substitute cardiac cells' loss shows a disorganized ECM fiber pattern at the different ventricular layers.^29^ Moreover, the fibrotic scar is characterized by progressive disruption of the viscoelasticity at different scales. The ECM fibers, measured by AFM, show increased viscoelastic properties compared to healthy ECM.^25^ These changes are translated to a general stiffening at the tissue and ventricular level that impairs muscle contraction and relaxation.^30,31^

## Cell Models to Mimic Cardiac Pathophysiology

The healthy adult mammalian heart comprises various cell types. The most functionally prominent ones are cardiomyocytes, smooth muscle cells, endothelial cells, and fibroblasts. In terms of cellular composition, it has been observed that the adult murine myocardium is composed of 56% cardiomyocytes, 27% fibroblasts, 7% endothelial cells, and 10% cardiac smooth muscle cells.^32^ Although precise numbers for human myocardial composition are unknown, similar ratios are proposed.^33^ With regards to the contractile function of the heart, the cardiomyocytes are the central cell type. Under physiological conditions, the endothelial cells are only involved as structural components of blood vessels, cardiac metabolism, and angiogenesis; the fibroblasts regulate the connective tissue with a primary role in producing the ECM; and the cardiac smooth muscle cells are involved in regulating the blood flow in the cardiac vasculature.^34^

The development of *in vitro* cardiac models relies on cell sources that accurately recapitulate human (patho)physiological phenotypes. Primary cells derived from rodents have been treated with various stimuli (i.e., pharmacological agents, culture substrate stiffness, and regulation of gene expression) to induce a variety of cardiovascular diseases.^35–38^ However, animal-derived cells show differences in molecular mechanisms compared to humans, limiting their translational potential.^39^ Human primary cells have also been derived from patients with or without the disease of interest. Although primary cells are a good representative of the tissue that they are derived from, they show display donor-to-donor differences, are challenging to obtain, show slow proliferation and limited life span, and rapidly dedifferentiate after isolation.^40,41^

The generation of induced pluripotent stem cells (iPSCs) more than one decade ago has emerged as a promising alternative cell source. Human iPSC (hiPSC) can be derived from somatic cells of patients that self-renew at a high rate and indefinitely and circumvent the ethical issues related to primary and embryonic stem cells. Moreover, they show little differences between batches and allow high reproducibility between studies.^42^ In addition, these cell-based models have been used to study the onset of heart failure *in vitro* with predetermined genetic mutations, including hypertrophic cardiomyopathies (i.e., mutation in *MYH7*),^43^ dilated cardiomyopathies (i.e., mutation in cardiac *Troponin T* or *PLN*),^44,45^ arrhythmogenic cardiomyopathies (i.e., mutation in *RyR2*), and channelopathies (i.e., mutation in *KCNH5*).^46,47^ Although cardiomyocytes derived from hiPSCs have revolutionized cardiac *in vitro* studies, they still lack the mature contractile, electrophysiologic phenotype reminiscent of adult cells.^48,49^

The lack of physiological maturity of hiPSC derived cardiomyocytes remains one of the greatest caveats of this cell source in modeling cardiac diseases. However, due to their great promise as the ideal cell source for modeling cardiac diseases, great efforts are being placed on improving the overall maturation of hiPSC derived cardiomyocytes.^50^ How mechanical cues affect hiPSC maturation is extensively discussed elsewhere.^51,52^

Physiologically, cardiomyocyte behavior is strongly influenced by the presence of fibroblasts. Banerjee *et al.* found that the direct contact between cardiomyocytes and fibroblasts *in vitro* leads to a decrease in the contraction velocity of cardiomyocytes.^53^ The fibroblast stimulation with pro-fibrotic factors to induce fibroblast phenotype characteristic of stiff environments has also been observed to promote a hypertrophic phenotype in cardiomyocytes.^54,55^ Therefore, to model the (patho)physiological setting of the heart, it is essential to consider the various cell types present within the tissue and the effects these may have on the mechanical properties of the tissue.

## Two-dimensional *In Vitro* Modeling of Cardiac Mechanobiology

Standard cell cultures in plastic dishes are not representative of the physical environment present in the heart. Therefore, more reliable cell models must be integrated into platforms mimicking the mechanical cues present in the *in vivo* microenvironment. Two-dimensional *in vitro* models can be particularly useful because they enable researchers to subject cells, in particular cardiomyocytes, cardiac fibroblasts, or cocultures thereof, to different mechanical cues such as (dis)organization, ECM stiffness, and external loads in a controlled and precise manner and without extreme experimental complexity. Moreover, these models allow for single cell analyses and the study of cell–cell interactions because they are easily assessable by microscopy and techniques like protein patterning, which are optimized for, or even restricted to, 2D models.

**FIG. 2.**
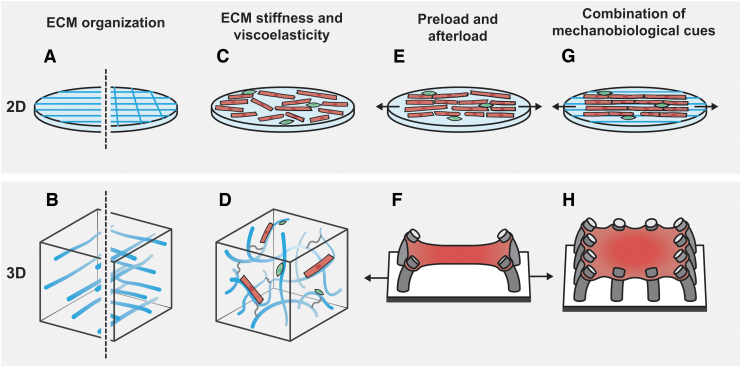
Examples of 2D and 3D cardiac *in vitro* models to mimic mechanobiological cues. **(A, B)** Two-dimensional patterned substrate with ECM proteins and 3D scaffold to model healthy (anisotropic, *left*) and diseased (isotropic, *right*) cardiac ECM. **(C, D)** ECM stiffness and viscoelasticity can be tuned in 2D and 3D environments using synthetic, natural, or hybrid polymers. Cardiomyocytes (*red*) and cardiac fibroblasts (*green*) can be cocultured on top of or inside a hydrogel network. **(E, F)** Static mechanical loading (*arrows*) can be used to control preload in 2D and 3D systems. In the EHT, afterload can be manipulated by changing flexible posts' stiffness thereby increasing resistance to cardiomyocyte contraction. **(G)** Mechanobiological cues can be combined in 2D by ECM patterning on top of a hydrogel of tunable stiffness under mechanical loading. **(H)** Van Spreeuwel *et al.* presented a 3D EHT model^94^ in which ECM organization can be controlled by geometrical constraints. Combining this technique with a hydrogel material with tunable stiffness would allow the study the combined effect of these mechanobiological cues. 2D, two-dimensional; 3D, three-dimensional; ECM, extracellular matrix; EHT, engineered heart tissue.

To mimic the (dis)organization of cardiac cells in healthy and diseased myocardium, microcontact printing is often used to pattern (an)isotropic substrates with a variety of proteins, including fibronectin,^35,48,56–58^ laminin,^56^ collagen,^59^ Matrigel,^60^ and gelatin,^61^ to force cells to adopt a specific shape, direction, and alignment (Fig. 2A). Another approach to induce anisotropic organization is to introduce contact guidance cues such as microgrooves^62^ in the substrate. In anisotropic organization, an obvious consequence of cell alignment is that cells become elongated with more aligned actin,^58^ myofibrils, and Z-lines,^48,60^ which correlate with an increased contraction force in both hiPSC-derived^60^ and primary rat cardiomyocytes.^48^ Moreover, enforcing an elongated shape on cardiomyocytes also increases their maturity in terms of electromechanical coupling.^60^ Since current 2D *in vitro* cardiac models successfully model the (an)isotropy of cardiac tissue, future disease models should also address the dynamics of reorganization to understand how the myocardium reorganizes under pathological conditions and whether this disorganization is reversible.

To simulate the stiffness of physiological (10–15 kPa) and fibrotic (30–100 kPa)^63^ cardiac ECM, cells in 2D models are typically cultured on protein-coated elastic hydrogels such as polyacrylamide (PAA)^57,58,60,61^ and polydimethylsiloxane (PDMS)^64–66^ gels (Fig. 2C). It has been demonstrated that culturing hiPSC-derived cardiomyocytes on substrates with a physiological stiffness improves both their maturation^67^ and contractile function,^60^ whereas increasing substrate stiffness to mimic that in a fibrotic environment reduces cardiomyocyte contractile force,^58,60,68^ sarcomere shortening,^68^ and myofibril integrity.^60^ One of the shortcomings of PAA and PDMS substrates is that they can only simulate cellular environments with constant mechanical properties, whereas *in vivo* ECM mechanics are dynamic due to the continuous remodeling of the ECM by cells, especially under pathological conditions. To address this limitation, PDMS substrates with magnetic inclusions^66^ and methacrylated hyaluronic acid gels^69^ have been developed, which can be stiffened on demand using a magnetic gradient or ultraviolet irradiation, respectively.

When using fully crosslinked linear elastic substrates such as PAA, the viscoelasticity of cardiac tissues^70^ is not accounted for. Nonfully crosslinked PAA gels, on the other hand, show viscoelastic behavior. Varying the concentrations of acrylamide (monomer) and bisacrylamide (crosslinker) allows the formation of gels with equal stiffness but variable viscoelastic properties.^71^ Since cardiomyocytes are particularly sensitive and responsive to viscoelasticity,^72^ further efforts are required to design materials with precisely tunable viscoelastic properties.^73^

Another essential component of the myocardium's mechanobiological environment studied using 2D models is the external load to which the myocardium is subjected. In several models, cardiac cells are cultured in monolayer on stretchable membranes in custom-built^59,74,75^ or commercially-available^62,76^ devices that can apply uni- or biaxial strain in a static or dynamic manner (Fig. 2E). Applying static strain provides a means to model different degrees of preload (i.e., sarcomere length of cardiomyocytes), whereas a dynamic strain is often used to simulate the cyclical loading that cardiomyocytes experience from the adjacent myocardium. Regardless of the stretching mode, changes in the proteins involved in gap junctions and cell–cell connections such as connexin 43 and N-cadherin are reported in response to stretch.^59,75^ Interestingly, the alterations observed in terms of electrophysiology and force transmission resulting from cell stretching in 2D *in vitro* show similarities to pathological remodeling associated with arrythmia^77,78^ and heart failure.^74^

Table 1 summarizes the strengths and limitations of 2D *in vitro* models developed to simulate various cardiac mechanobiological cues. Of these, only a few allow the integration of multiple mechanobiological cues (Fig. 2G) in combination with functional readouts, such as cell force and electrical activity. In addition, most models assume mechanobiological cues to be constant and therefore fail to capture the dynamic nature of these cues *in vivo*. Future efforts should address these limitations.

**Table 1.** Summary of Strengths (++) and Limitations (− −) of Relevant Two-Dimensional and Three-Dimensional Cardiac *In Vitro* Models That Have Been Developed to Simulate Various Mechanobiological Cues.

Models	Mechanobiology aspects						Readouts				References
	Dimensionality	Stiffness	Viscoelasticity	ECM organization	Mechanical stretch	Number of cells needed	High throughput	Contraction	Electrical activity	Calcium dynamics	
Two-dimensional											
I. ECM-patterned PAA/PDMS substrates	− −	++	− −	++	− −	++	+	++	++	++	^58,60^
II. Microgroove PDMS substrates	− −	++	− −	+	− −	++	+	+	++	++	^62^
III. PAA substrates	− −	++	− −	− −	− −	++	++	++	++	++	^60,61^
IV. PDMS substrates	− −	++ D^52^	− −	− −	− −	++	+	+	++	++	^64,65^
V. Strained membranes	− −	−	− −	− −	++ D	++	− −	+	+	+	^62,76^
Three-dimensional											
I. Free-floating hydrogel constructs	++	++	+	−	− −	− −	−	++	+	+	^80,81^
II. 3D scaffolds	++	+	− −	++	++	− −	−	+	++	++	^86,125,126^
III. EHTs	++	+	−	+	++ D	− −	−	++	++	++	^88,94,127^

D indicates the dynamic tunability of the mechanobiological cue in the highlighted model.

3D, three-dimensional; ECM, extracellular matrix; EHTs, engineered heart tissues; PAA, polyacrylamide; PDMS, polydimethylsiloxane.

## Three-dimensional *In Vitro* Modeling of Cardiac Mechanobiology

To better recapitulate the 3D complexity of cardiac mechanobiology, there is a considerable research interest in 3D *in vitro* model systems that mimic the complex 3D architecture and mechanics of the myocardium. By means of biomaterials, external loading, or tissue processing techniques, it is possible to include the mechanical cues from the anisotropic and continually-beating cardiac tissue in 3D *in vitro* models.

Over the past decades, many scaffold materials have been explored to mimic the stiffness and 3D architecture of myocardial ECM. Hydrogels composed of crosslinked polymer networks such as gelatin methacryloyl, polyethylene glycol (PEG), and alginate are regularly used, of which the elastic modulus can be tuned in the range of physiological and pathological myocardium.^79–83^ The usage of these hydrogels is often preferred over hydrogels that are reconstituted from natural ECM such as collagen, fibrin, basement membrane, or decellularized ECM, owing to the possibility to control the physical and mechanical properties systematically. However, hydrogels composed of crosslinked polymer networks often lack the mechanosensitive ligands present in native ECM, and chemical crosslinking of polymers can negatively affect the remodeling capacity of encapsulated cells.^84^

To take advantage of the interplay between crosslinked polymer networks and natural polymers, hybrid biomaterials have been developed, such as poly(lactic-*co*-glycolic acid)/gelatin/elastin and poly (caprolactone) (PCL)/gelatin.^85^ Moreover, material processing techniques, such as melt-based electrohydrodynamic printing and electrospinning, are used to mimic the hierarchical microarchitecture of the myocardium in 3D scaffold materials (Fig. 2B). Lei *et al.* printed PCL microfibers together with submicro scale conductive fibers in a layer-by-layer manner to guide layer-specific cellular alignments, demonstrating the applicability of such processing techniques to fabricate multifunction microarchitectures with biomimetic architectures.^86^

The viscoelasticity of the natural ECM has been implemented by the use of hydrogel materials (Fig. 2D). However, there are only a few materials that allow controlled tuning of the viscoelasticity. Some recent studies show that viscoelasticity can significantly affect cellular behavior and specifically mechanosensing. Chaudhuri *et al.* modulated the nanostructure of alginate-based hydrogels to develop materials with a wide range of stress relaxation rates but a similar elastic modulus. They showed that stress relaxation affects actomyosin contraction and mechanical clustering of adhesion ligands, highlighting that stress relaxation is a crucial player in controlling cell-ECM interactions.^72^ Moreover, a recent study by Ma *et al.* used a fast-relaxing boronate-PEG hydrogel system to study the effects of viscoelasticity on cardiac myofibroblast activation. They found that the spreading and activation state of the myofibroblasts were significantly higher in high viscoelastic hydrogels, mimicking the viscoelasticity of native myocardium, as opposed to medium and low viscoelasticity.^87^ Collectively, these studies show that viscoelasticity, although its importance is often overlooked, can significantly influence how cells sense the mechanical cues from their environment.

To incorporate the mechanics of preload and afterload in 3D *in vitro* models, Eschenhagen *et al.* pioneered the design of engineered heart tissue (EHT).^88^ This cardiac microtissue was composed of a cell-laden, ECM mimicking hydrogel molded between two stretching posts. The posts were used to maintain mechanical stretch of the tissue, and hence the initial prestretch of the sarcomeres, and simultaneously functioned to quantify contractile forces exerted by the tissue (Fig. 2F). To date, EHTs serve as the gold standard to model the mechanical behavior of myocardial tissue; although fabrication protocols vary among studies, each design incorporates its own method to mechanically stimulate the tissue. Boudou *et al.* miniaturized the EHTs for high-throughput experiments and adapted the design to independently vary either the mechanical stiffness of the stretching posts or the mechanical stiffness of the ECM mimicking hydrogel. They showed that both the cardiac contraction force and the basal static tension within the EHTs improved with increasing preload and ECM rigidity.^89,90^

By changing the static prestretch of the EHT constructs preload can be varied.^91^ By incorporating their EHT system in a Flexcell device to vary the preload of engineered tissues, van Kelle *et al.* found that tissue contractile force increased with increasing preload.^92^ Abilez *et al.* molded EHTs between a rigid post and a flexible post. The resistance to contraction of the flexible post could be manipulated to adjust the afterload. By combining their experiments with computational modeling, they assessed the effects of passive stretch on the structural and functional maturation of EHTs and optimized the cell alignment and calcium dynamics within EHTs, providing a basis for the rational design of EHT parameters.^93^

Overall, the EHT approach enables independent control over mechanical cues applied to (e.g., stretch) and inside (e.g., hydrogel properties) the microtissues. Classical EHTs, however, are confined in one direction to mimic the myocardium's anisotropy, which is not representative of most pathological tissues. To mimic the pathological, chaotic tissue organization and mechanical force distribution of the myocardium, *in vitro*, Van Spreeuwel *et al.* demonstrated the fabrication of cardiac microtissues consisting of cardiomyocytes and fibroblasts that could be constrained either uniaxially and biaxially, thereby manipulating the organization and mechanical forces within the microtissue while simultaneously quantifying the contraction force exerted by the tissue (Fig. 2H).^94^ Mimicking pathological relevant cell ratios and ECM properties in such biaxially constrained EHTs will greatly benefit the development of disease models that mimic the myocardial microenvironment upon injury.

More recently, researchers have put effort into better recapitulating ventricle anatomy and function *in vitro*. By means of advanced 3D bioprinting and electrospinning techniques the characteristic ECM fiber anisotropy has been mimicked while building a left ventricle chamber of few millimeters.^95,96^ The scaffolds were seeded with iPSC derived cardiomyocytes and showed functional electrophysiological activity leading to contraction and, even, ejection fraction.^96^

While great advances have been made in developing *in vitro* myocardial microtissues, there is a lack of physiological microtissues that incorporate the complete mechanical signature of the healthy and diseased myocardial microenvironment. Relevant 3D methods to mimic mechanobiological cues for cardiac *in vitro* tissue modeling are summarized in Table 1.

## Outlook and Outstanding Challenges

**FIG. 3.**
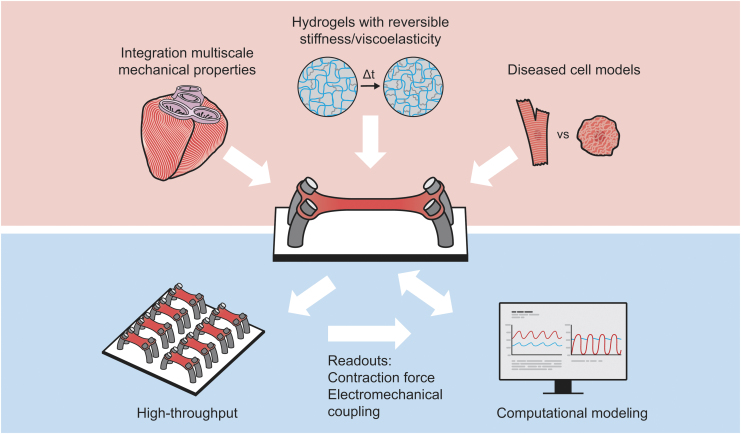
Outstanding challenges for the state-of-the-art *in vitro* cardiac tissue models to improve our knowledge of cardiac mechanobiology. To better mimic the most relevant aspects of the cardiac environment, the next generation models require (1) the characterization and integration of both the mechanical and architectural properties of cardiac tissue from cell to tissue scale, (2) hydrogels with tunable and reversible mechanical properties to mimic dynamic changes in cardiac pathology, and (3) utilize genetically-predisposed diseased cells. Moreover, the models should enable high-throughput experiments to increase experimental reproducibility and statistical readout reliability (including contraction force and electromechanical readouts). Ultimately, the readouts could be integrated into computational models to unravel the role of mechanobiological cues in cardiac (patho)physiology and predict long-term results, thereby increasing the translational potential.

In this section we highlight some of the outstanding challenges that cardiac *in vitro* models are facing to improve our knowledge of cardiac mechanobiology in health and disease (Fig. 3) and the most important drawbacks of current engineered tissue models in light of understanding cardiac tissue mechanobiology.

### Integration of multiscale cardiac mechanical properties

To be able to understand the effect of physical tissue properties on cell and tissue behavior, it is essential to characterize these under healthy and diseased conditions. To this end, one of the key challenges to develop accurate *in vitro* cardiac tissue models is to precisely characterize the mechanical environment of the myocardium, both at the cellular and tissue level. One of the great hurdles in this aspect is our lack of knowledge regarding the passive mechanical properties (i.e., stiffness and viscoelasticity) in the human myocardium.^20^ In addition, although the strain present *in vivo* in the myocardium has been reported using ultrasound and magnetic resonance imaging, these techniques show relatively low resolution at cellular scale.^97,98^

To develop *in vitro* cardiac disease models, myocardial mechanical properties upon cardiac injury need to be characterized. For example, after MI, there is a pro-inflammatory phase followed by myocardial tissue remodeling.^99^ Several approaches try to restore cardiac functionality at this phase before the fibrotic scar is formed.^100^ However, there is still a lack of knowledge of how inflammation affects ECM turnover and the overall myocardial mechanical properties. The characterization of the mechanical properties of human myocardium from the cell niche to tissue level will be the first step toward integrating these mechanical factors into the future cardiac tissue *in vitro* models (Fig. 3).

### Hydrogels with reversible mechanical properties

To mimic the dynamic mechanical properties in cardiac diseases, the hydrogels need to change their properties in time (Fig. 3).^101^ In addition, the level and nature of crosslinking in the polymeric hydrogel network greatly define the viscoelastic properties of the hydrogel.^102^ Standard hydrogels are based on covalent crosslinking between structural polymeric fibers to define their mechanical properties. By incorporating reversible crosslink methods, the hydrogel can change its properties when an external trigger such as temperature, light, or chemical agent is applied.^103,104^ For example, Wu *et al.* used a PEG hydrogel modified with a photo-sensible reversible crosslinker that allowed to dynamically tune the viscoelastic properties by use of blue light. Most notable is that the changes were even possible when culturing cells inside the hydrogel.^105^ A more recent advancement was made with the development of pH sensible hydrogels, which enables the changing of the degree of crosslinking by dynamically changing the pH in the polymeric network.^106^

While these hydrogels show great promise, they are based on synthetic polymeric materials and, therefore, lack the presence of biological cues, which are offered by naturally derived hydrogels. Thus, the ultimate goal would be to develop hydrogels with the reversible tunable mechanical properties while maintaining biological relevance for cardiac cells.

### Diseased cell models

The discovery of hiPSCs more than a decade ago and the development of differentiation protocols for numerous cell types, including cardiomyocytes and fibroblasts, have strongly improved our ability to model cell (patho)physiology *in vitro*. One of the advantages of hiPSCs is that these cells can be obtained from genetically predisposed diseased patients to study specific cardiovascular diseases.^107^ The use of hiPSC-derived cardiomyocytes with a diseased phenotype combined with a diseased mechanical environment can reveal the role of mechanobiology in cardiac pathophysiology (Fig. 3).

The interaction between cardiomyocytes and the other cell types present in the myocardium is of utmost importance to understand tissue behavior. This is highlighted by studies that found that fibroblast phenotype and fibroblast–cardiomyocyte interaction considerably influence the internal cardiomyocyte structure, contractile machinery, and electrophysiology in cardiomyocyte and fibroblast cocultures in 2D and 3D *in vitro* models.^108–111^ Importantly, the physical environment also influences fibroblast behavior.^112^ Therefore, the interplay between cardiomyocytes, other cardiac cell types, and the mechanical environment remains an underexplored area that is instrumental in understanding cardiac (patho)physiology.

### Electromechanical coupling

Coordinated contraction of the heart and heart rhythm maintenance is controlled by a highly interconnected cardiomyocyte network that communicates through gap junctions between neighboring cells connected in series. Cardiomyocytes have autorhythmic properties. At the tissue level, abnormalities in electrical coupling—for instance due to nonaligned cells or necrotic cells—will lead to a locally desynchronized contraction or arrhythmias.

Cardiac *in vitro* tissues containing mature cardiomyocytes typically start self-beating a few hours after cell seeding.^94^ However, usually these tissues beat nonsynchronously due to a lack of proper cardiomyocyte and fibroblast organization and cell–cell contact inside the hydrogel.^111^ To overcome this problem, cardiac *in vitro* tissues are commonly electrically paced during long-term culture.^113^ Moreover, it is also recognized that mechanobiological features of the cellular environment can affect cardiomyocyte electromechanical coupling. In particular, the ECM elastic modulus and dynamic stretch can alter the pattern of calcium transients and cell–cell connections.^114,115^ However, little is known about how these features eventually affect tissue contractile properties, for instance during adverse remodeling of cardiac tissue, but *in vitro* cardiac tissue models may shed light on this topic in future studies.

### High-throughput technologies

Automated high-throughput technologies of cardiac tissue *in vitro* models are desired to increase experiment reproducibility and statistical reliability, for example, in drug screening (Fig. 3). The development of EHTs involves the use of complex microfabrication techniques, limiting its repeatability.^14^ In addition, they demand a high number of cells compared to standard 2D models. Some efforts have been made to miniaturize EHTs, increasing the number of devices per culture plate and reducing the amount of cells needed.^116^ This miniaturization has a drawback that the *in vitro* tissue loses dimensionality (i.e., the hydrogel is only a few micrometers thick) while still requiring complex fabrication techniques.

Recently, the rapidly expanding possibilities of 3D bioprinting make this an appealing technique to increase the throughput of 3D *in vitro* cardiac tissue models. In this sense, the development of magnetic, electrically conductive, and piezoelectric bioinks could serve as an alternative to EHT platforms to quantify tissue contraction and electrophysiology and apply mechanical stretch to the 3D culture at the same time.^117,118^

In terms of readouts, currently, most of the techniques are limited to standard 2D models due to microscopy requirements. Therefore, their applicability in the 3D models is not feasible or very limited. For instance, 3D traction force microscopy has been developed to measure cell-ECM force in 3D setups, but its high imaging and computational complexity makes its applicability limited to specialized laboratories.^119^ Thus, the improvement and development of new tools are needed to facilitate readouts in complex *in vitro* 3D models.

### Computational modeling

Nowadays, multiscale cardiac computational models are able to link cellular electrophysiology, myocardial tissue mechanics, and cardiac hemodynamics that serve to simulate and predict heart function and evaluate novel diagnostic and therapeutic strategies. The reader is referred to recent publications tackling these models.^120–122^

In terms of cardiac *in vitro* models, computational models should allow to describe and predict how mechanobiological cues like stiffness, viscoelasticity, ECM organization, and tissue strain affect tissue contraction and electrophysiology (Fig. 3).^123^ When used to predict such parameters under pathological conditions, the computational models should describe how cardiac cells, cell–cell interactions, and ECM structurally, mechanically, and electrically remodel in response to pathophysiological stimuli.

The added and complementary value of computational models further lies in the fact that they allow to deconvolve the effect of each mechanobiological factor rationally. In addition, almost all experimental knowledge of how mechanical factors affect cardiac cell behavior are restricted to single cell or cell monolayers in 2D models. The use of computational models will be crucial to link cell behavior in 2D to the more complex 3D morphology and shape of 3D tissue models and eventually may be used to predict whole heart functionality. Moreover, *in vitro* models are restricted to experiments that last days or, at most, few weeks, limiting the understanding of long-term effect of mechanobiological factors on tissue behavior. In this sense, computational models can also help to rationally predict the long-term results improving the translational applicability of the engineered *in vitro* models.^124^

### Limitations

Although cardiac *in vitro* models show a great potential to study cardiac mechanobiology, they are still far from replicating human physiology. The abovementioned challenges are a clear example of the enormous efforts that still have to be made to better mimic cardiac environment and function. Limitations in iPSC derived cardiomyocyte maturation, lack of spatial cell organization, cell diversity, and reliable electrophysiology still limit *in vitro* models' potentiality. Moreover, *in vitro* models cannot resemble the complexity of the whole organism and cross talk between organs and systems. One clear example of the cross talk between systems is the immune system, essential for tissue homeostasis and remodeling after injury. Therefore, *in vitro* models are essential to systematically study mechanobiological environment but cannot replace the translational potential of *in vivo* models.

## Conclusions

In summary, cardiac *in vitro* models are powerful platforms to quantify the functional impact of mechanobiological cues in cell and tissue behavior. This review has discussed the state-of-the-art knowledge and methods to mimic cardiac mechanobiology in 2D and 3D setups. Two-dimensional cardiac *in vitro* models have the advantage to precisely control the mechanobiological cardiac cues in terms of ECM stiffness, organization, and cyclic strain. The challenge is to translate the knowledge gained from these models to develop 3D models with the ability to precisely tune the cardiac environment. In this sense, it is fundamental to develop hydrogels to mimic the ECM organization and viscoelastic properties at different scales. Moreover, the combined use of genetically predisposed diseased cardiomyocytes, cardiac fibroblasts, and a diseased ECM environment will strengthen pathology mimicking models. At the same time as gaining model complexity, the development of new tools to facilitate readouts in 3D, together with the use of computational models, will enable to improve also the mechanistic understanding of cardiac mechanobiology. An interdisciplinary approach, including hydrogel development, new fabrication techniques, together with clinically relevant cell models, is essential for the progress of this field.
